# Comparative effects of high-intensity and sprint interval training on cardiorespiratory fitness and body composition: a systematic review with meta-analysis

**DOI:** 10.3389/fphys.2025.1668326

**Published:** 2025-11-11

**Authors:** Da Zhang, Jie Dong, Chien-Wen Hou, Jie-ping Wang

**Affiliations:** 1 School of Physical Education, Quanzhou Normal University, Quanzhou, Fujian, China; 2 School of Education, University of New South Wales, Sydney, NSW, Australia; 3 Laboratory of Exercise Biochemistry, University of Taipei, Tianmu Campus, Taipei, Taiwan; 4 School of Exercise and Health, Shanghai University of Sport, Shanghai, China

**Keywords:** body fat percentage, obesity, HIIT, SIT, physical fitness

## Abstract

**Background:**

Interval training modalities, including high-intensity interval training (HIIT) and sprint interval training (SIT), are widely recognized for their efficiency and health benefits. However, it remains unclear how baseline fitness levels influence the differential effects of HIIT and SIT on key health-related outcomes.

**Objective:**

This meta-analysis aims to compare the effects of HIIT and SIT on cardiorespiratory fitness (CRF) and body fat mass, with subgroup analyses based on participants’ health and training status.

**Methods:**

Nine randomized controlled trials (n = 666) were included. Primary outcomes were changes in VO_2_max/VO_2_peak and body fat percentage. Data were synthesized using standard mean difference (SMD) or weighted mean difference (WMD), with subgroup analyses stratified by population type (healthy/trained vs. overweight/obese). This review was registered in PROSPERO (CRD420251016362; registered on 15 March 2025).

**Results:**

Both HIIT and SIT significantly improved CRF (SMD = 1.54; 95% CI: 0.89–2.18; p < 0.00001) and reduced body fat mass (WMD = −3.45%; 95% CI: −5.04 to −1.87; p < 0.0001) compared to control. Subgroup analyses revealed that HIIT was more effective in improving CRF in overweight/obese individuals (SMD = −0.97; p = 0.0004), while SIT was more effective in reducing fat mass among healthy or trained populations (WMD = 5.85; p < 0.00001).

**Conclusion:**

Both HIIT and SIT are effective interventions for enhancing CRF and reducing body fat, but their relative efficacy may depend on participants’ baseline health status. HIIT appears optimal for individuals with lower fitness levels, while SIT may be preferable for time-efficient fat reduction in trained populations. Tailoring interval training prescriptions to individual characteristics is recommended.

**Systematic review registration:**

https://www.crd.york.ac.uk/PROSPERO/view/CRD420251016362.

## 1 Introduction

Physical fitness is widely recognized as a critical indicator of overall health status. It is generally composed of four key components: cardiorespiratory fitness (CRF), musculoskeletal fitness, motor fitness, and body composition (Pinho et al., 2024). Among these, CRF and body composition are considered core indicators of health-related physical fitness. They are not only closely associated with athletic performance but are also widely utilized in chronic disease risk assessment (Bull et al., 2020). Epidemiological evidence has demonstrated that higher levels of CRF are significantly associated with lower risks of all-cause mortality and cardiovascular events. Specifically, each 1-MET increase in maximal aerobic capacity is linked to a 13% reduction in all-cause mortality (Kodama et al., 2009). In addition, elevated fat mass is a major risk factor for a wide range of chronic diseases, including type 2 diabetes, cardiovascular disease, and several types of cancer. It is also associated with reduced quality of life, sleep disturbances, and impaired physical function (Murray et al., 2020).

In recent years, interval training has attracted considerable attention due to its time efficiency and physiological benefits. Among its modalities, High-intensity interval training (HIIT) and sprint interval training (SIT) have emerged as two widely studied forms. HIIT typically involves repeated bouts of high-intensity exercise interspersed with active or passive recovery periods. According to Buchheit and Laursen, HIIT protocols can be broadly categorized into SIT—defined as repeated “all-out” sprints lasting ≤30 s with 2–4 min of recovery—and other submaximal HIIT formats (Buchheit and Laursen, 2013). However, there remains no universal consensus on the definitions of HIIT and SIT in the literature. For instance, some studies characterize SIT as ≤45-s all-out efforts regardless of duration, while HIIT is generally performed at high but submaximal intensities (Boullosa et al., 2022). Consequently, accurately determining the power output during training is essential when prescribing HIIT protocols. Although HIIT and SIT share similar interval structures, they differ in metabolic stress, recovery demands, and exercise tolerance. Directly comparing these modalities may help clarify their relative physiological and practical advantages.

Furthermore, individuals with different baseline fitness levels may exhibit divergent responses to interval training. Healthy or trained populations generally possess higher initial cardiorespiratory fitness and metabolic flexibility, which may influence their adaptability to various training stimuli (Garthwaite et al., 2024). In contrast, individuals with overweight or obesity often present with lower baseline CRF, impaired fat oxidation, and greater cardiometabolic risk, potentially leading to distinct physiological adaptations (Amaro-Gahete et al., 2019). Reduced fat oxidation capacity is closely linked to impaired metabolic flexibility, increased lipid accumulation, and insulin resistance, which together contribute to higher risks of metabolic and cardiovascular disorders (Cocks et al., 2016). Recent studies suggest that HIIT is particularly effective in improving VO_2_max in individuals with low baseline fitness or obesity, compared to healthy individuals (Wen et al., 2019). By comparison, SIT enhances fat oxidation efficiency with less total exercise time, making it a time-efficient option for healthy or trained individuals. Although previous meta-analyses have examined the general effects of HIIT and SIT, few have systematically evaluated how population characteristics such as baseline fitness status influence these outcomes.

While both HIIT and SIT have demonstrated efficacy across various outcomes, training responses may vary depending on baseline health status, training history, sex, and intervention design. Therefore, the present study conducts a systematic review and meta-analysis comparing the effects of HIIT and SIT on CRF and body composition, incorporating subgroup analyses based on population health characteristics. Unlike previous reviews that primarily compared each modality with moderate-intensity training, the present analysis provides a direct head-to-head comparison between HIIT and SIT. This approach aims to provide more nuanced and individualized guidance for exercise prescription.

## 2 Methods

### 2.1 Study protocol and registration

This systematic review and meta-analysis was conducted in accordance with the PRISMA 2020 guidelines, and the methodological framework adhered to the Cochrane Handbook for Systematic Reviews of Interventions (version 6.4) to ensure transparency and reproducibility. The present work has been registered to the International Prospective Register for Systematic Reviews (PROSPERO—registration number: CRD420251016362; registered on 15 March 2025), and the registration record is publicly available at: https://www.crd.york.ac.uk/PROSPERO/view/CRD420251016362.

A comprehensive literature search will be conducted across the following electronic databases: PubMed, Embase, Web of Science, Cochrane Library, and SPORTDiscus, from inception to the last search on 15 March 2025, with no language restrictions applied. The search will include all studies published up to the most recent date of the search, with no restrictions on the start date. A combination of keywords and Medical Subject Headings (MeSH) terms will be used, including but not limited to: “high-intensity interval training” OR “HIIT,” “sprint interval training” OR “SIT,” “cardiorespiratory fitness” OR “VO_2max_,” and “fat loss” OR “body composition” OR “body fat percentage.” Boolean operators (AND/OR) will be applied to refine the search strategy for each database. The complete, database-specific search strings, together with all applied filters and Boolean operators, are provided in Supplementary Table S1 to ensure transparency and reproducibility. All identified references will be imported into EndNote, and duplicates will be removed. Two reviewers will independently screen the titles and abstracts to exclude irrelevant studies, followed by a full-text review of potentially eligible articles based on the predefined inclusion and exclusion criteria. Any discrepancies between reviewers were resolved through discussion and consensus; when disagreement persisted, a third senior reviewer was consulted to make the final decision. Additionally, the reference lists of included studies will be manually screened to identify any additional eligible studies. The screening process will be documented and presented in a PRISMA flow diagram, including details on the number of studies identified, screened, excluded, and included, along with reasons for exclusion at the full-text stage.

### 2.2 Inclusion/exclusion criteria

The study was conducted following the PRISMA (Preferred Reporting Items for Systematic Reviews and Meta-Analyses guidelines. There were selected based on the Population, Intervention, Comparison, Outcomes and Study (PICOS) model (Table 1). Nine intervention studies were included. The primary outcome of the present meta-analysis included Vo_2max_ or Vo_2peak_, while the secondary outcome was fat mass percentage. Studies were eligible if they reported quantifiable measures of body composition (e.g., body fat percentage or fat mass), regardless of the measurement technique used (e.g., DEXA, bioelectrical impedance, or skinfold thickness). The measurement method was recorded but not used as an inclusion or exclusion criterion. Studies were excluded if: (a) duplicate publications; (b) there were not control group data; (c) did not include pre- and post-data about VO_2max_ or Vo_2peak_ and fat mass; (d) there was a mixed intervention (i.e., if involving medication or dietary changes); (e) there was a lack of detailed results and full-text data; or (f) only one of the two interventions (HIIT or SIT) was examined, as the present meta-analysis aimed to directly compare their relative effects under similar experimental conditions.

**TABLE 1:** Eligibility criteria of studies based on PICOS model.

Parameter	Inclusion criteria
Population	Healthy individuals
Intervention	HIIT and SIT at least 2 weeks
Comparators	Control
Outcomes	VO_2max_ or Vo_2peak_ (mL·kg^−1^·min^−1^) and fat mass (percentage) data
Study design	Only randomized controlled trials

### 2.3 Data extraction

All retrieved records were initially screened by the one investigator to assess eligibility based on predefined inclusion criteria. The screening results were then confirmed by at least two independent reviewers to minimize selection bias. References were managed using EndNote (Version 20; Clarivate Analytics, Philadelphia, PA, United States), where duplicate entries were removed and studies were screened through manual review. Once eligible studies were identified, relevant data were extracted and organized according to participant demographics (e.g., sample size, age, sex) and the type of exercise intervention (SIT, HIIT or control group). The outcome data were expressed as weighted mean difference (WMD) or standard mean difference (SMD).

### 2.4 Subgroup analysis

A subgroup analysis was performed to distinguish differential outcomes in physical fitness based on the type of intervention. Exercise interventions were categorized into HIIT and SIT. Physical fitness included cardiorespiratory fitness and body composition. Cardiorespiratory fitness was assessed using VO_2_max, while body composition was evaluated through body fat percentage. All VO_2_max/VO_2_peak values were expressed in mL·kg^−1^·min^−1^, and body fat outcomes were expressed as body fat percentage (%).

### 2.5 Quality assessment

The quality of the included studies was assessed using 5 domains according to the revised Cochrane Risk of Bias tool for randomized trials: (a) randomization process, (b) deviations from intended interventions, (c) missing outcome data, (d) measurement of the outcome, and (e) selection of the reported result. The overall risk-of-bias was defined as “low risk” if all domains were at low risk of bias, “some concerns” if containing at least 1 domain at some concerns status but not at high risk of bias for any domain, and “high risk” if at least 1 study was judged in some concerns for multiple domains.

### 2.6 Statistical analysis

Meta-analysis was performed using Review Manager (RevMan Version 5.4.1; Cochrane, London, United Kingdom). Due to the involvement of continuous data (mean ± SD), WMD or SMD with 95% confidence intervals (95% CI) were used to represent the final analysis. Means and SD from HIIT, SIT and control group data were collected. Subgroup analyses were performed for outcomes in relation to the HIIT or SIT. The Forest plots were as produced to display WMD, SMD, SD, and the overall effect of Z score. If publications reported standard error (SE) only, SD was calculated using the following formula, where n represented the number of participants: SD = SE*√n.

To assess the heterogeneity, tau-squared (*τ* ^2^), Chi-square Cochran’s Q (χ^2^) test, and *I* ^2^ statistic were performed. A value of *τ* ^2^ > 1 indicated variability between studies. Q test measured the variation around a weighted mean, in which p-value <0.10 was considered as significant heterogeneity. *I* ^2^ statistic was used to assess the effect consistency across the studies, with *I* ^2^ interpreted as follows: (a) *I* ^2^ = 0% to −29 30% showing no important heterogeneity, (b) *I* ^2^ = 30%–49% showing moderate heterogeneity, (c) *I* ^2^ = 50%–74% showing substantial heterogeneity, (d) *I* ^2^ = 75%–100% showing considerable heterogeneity (Bishop et al., 2019; Liu et al., 2025). Meta-analysis was performed using a fixed-effects model when heterogeneity was not significant (*I* ^2^ ≤ 50% and p ≥ 0.1) and a random-effects model was used when heterogeneity was significant (*I* ^2^ > 50% or p < 0.1). p-value <0.05 was considered statistically significant. Sensitivity analyses were performed in Review Manager (RevMan Version 5.4.1; Cochrane, London, United Kingdom) using a leave-one-out approach, whereby each study was sequentially excluded to examine its influence on the overall pooled effect.

## 3 Results

### 3.1 Literature search

#### 3.1.1 Selection process

**FIGURE 1:**
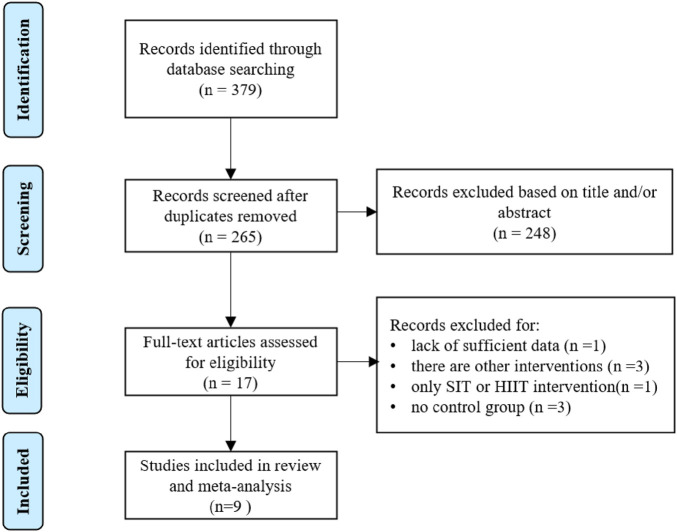
Process of elimination and inclusion of studies for review based on PRISMA guidelines.

The number of identified articles from four databases and selection process are shown in Figure 1. A total of 379 intervention studies were retrieved from the database search, and 362 duplicated and ineligible articles were excluded. The screening phase in this work, including title and abstract screening, left 17 articles. The authors excluded 8 articles from this meta-analysis due to: (1) lack of sufficient data (Oliveira et al., 2022); (2) there are other interventions (Cooke et al., 2022; Hsu et al., 2021; Hebisz et al., 2019); (3) only SIT or HIIT intervention (Salus et al., 2022); (4) no control group (Paquette et al., 2021; Hov et al., 2023; Sun et al., 2019). This screening resulted in 9 eligible articles that were used for the current quantitative analysis. These studies were included in systematic review (Esfarjani and Laursen, 2007; Inglis et al., 2024; Zhu et al., 2024; González-Gálvez et al., 2024a; González-Gálvez et al., 2024b; Hu et al., 2021; Tong et al., 2018; Schubert et al., 2017; Zhang et al., 2021).

#### 3.1.2 Quality assessment in individual studies

Among the included studies, no study scored in the high-risk bias, 3 studies scored in the moderate-risk bias (Inglis et al., 2024; Zhu et al., 2024; Hu et al., 2021), and 6 studies scored in the low-risk bias (Esfarjani and Laursen, 2007; González-Gálvez et al., 2024a; González-Gálvez et al., 2024b; Tong et al., 2018; Schubert et al., 2017; Zhang et al., 2021). The overall and domain-level results of the quality assessment are presented in Supplementary Figure S1 (traffic-light plot) and Supplementary Table S2 (domain-level summary). These results indicate that the overall methodological quality of the included studies was acceptable and that no major sources of bias were identified. Study Characteristics The characteristics of the included studies are summarized in Table 2 (Esfarjani and Laursen, 2007; Inglis et al., 2024; Zhu et al., 2024; González-Gálvez et al., 2024a; González-Gálvez et al., 2024b; Hu et al., 2021; Tong et al., 2018; Schubert et al., 2017; Zhang et al., 2021). All studies involved young adult participants aged between 13 ± 1 to 29 ± 8 years, including both healthy/trained individuals and those classified as overweight or obese. A total of 9 randomized controlled trials were analyzed, comprising participants across different fitness levels. The interventions were categorized into HIIT and SIT, with training durations ranging from 4 to 12 weeks.

**TABLE 2:** Study characteristics for HIIT and SIT interventions.

Study	Sample size and sex		Population characteristic (mean ± SD)			Exercise protocol							Key findings
			Age (y)	Activity status	VO_2max_/VO_2peak_ (mL/kg/min)	Period (weeks)	Type	Mode	Intensity	Repeats	Duration (s)	Rest (s)	
Esfarjani and Laursen (2007)	6 males		19 ± 2	Moderately trained	51.7 ± 3.4	10	SIT	Running	130% vVO_2max_	12	30	270	HIIT can improved VO_2max_.
	6 males				51.3 ± 2.4		HIIT		60% T_max_ at vVO_2max_	8	60% of Tmax	60% of Tmax	
	5 males				51.8 ± 2.8		Control	No exercise					
Inglis et al. (2024)	14 (7 females)		28 ± 6	Healthy	40.9 ± 6.6	12	SIT	Cycling	All-out effort	3–6	30	270	HIIT was more effective than SIT.
			27 ± 8		40.2 ± 6.2		HIIT		≥120% MMSS	6	240	180	
			27 ± 5		42.7 ± 6.0		Control	No exercise					
Zhu et al. (2024)	10 females		21 ± 2	Obese	27.8 ± 2.8	12	SIT	Cycling	All-out effort	40	6	9	Both SIT and HIIT decreased fat mass in obese women.
	10 females		20 ± 2		27.2 ± 4.2		HIIT		120% VO_2max_	16–21	60	90	
	10 females		20 ± 1		28.6 ± 2.5		HIIT		90% VO_2max_	5–7	240	180	
	9 females		21 ± 2		30.2 ± 3.1		Control	No exercise					
González-Gálvez et al. (2024a)	13 adolescents		13 ± 1	Healthy	23.5 ± 1.1	8	SIT	Runing	90–95%RHR	6	60	60	HIIT was more effective than SIT for improving fitness, blood parameters, blood pressure, and body composition.
	17 adolescents				23.9 ± 0.9		HIIT		80–85%RHR	3	120	120	
	18 adolescents				21.4 ± 0.9		Control	No exercise					
González-Gálvez et al. (2024b)	9 (3 females)		13 ± 1	Overweight	23.2 ± 1	8	SIT	Running	90–95% RHR	6	60	60	HIIT improved body composition, HR, and cardiorespiratory fitness; SIT only decreased fat mass.
	11 (5 females)				22.8 ± 1		HIIT		80–85% RHR	3	120	120	
	12 (6 females)				20.6 ± 1		Control	No exercise					
Hu et al. (2021)	15 females		21 ± 1	Overweight and obese	30.8 ± 3.7^a^	12	SIT	Cycling	All-out effort	80	6	9	SIT achieved similar improvements in VO_2peak_ and body composition as HIIT, with less time commitment.
	15 females		22 ± 2		31.6 ± 2.2^a^		HIIT		90% VO_2peak_	9	240	180	
	15 females		21 ± 1		28.8 ± 3.6^a^		Control	No exercise					
Tong et al. (2018)	16 females		21 ± 1	Obese	30.7 ± 3.5	12	SIT	Cycling	All-out effort	80	6	9	SIT enhanced time efficiency over traditional HIIT while effectively reducing abdominal visceral fat in obese young women.
	16 females		21 ± 1		30.2 ± 4.4		HIIT		90% VO_2max_	--	240	180	
	14 females		21 ± 2		28.6 ± 3.1		Control	No exercise					
Schubert et al. (2017)	30 (17 females)	12	29 ± 8	Moderately active	31.4 ± 9.2	4	HIIT	Cycling	90% PPO	6	60	60	SIT was more effective at increasing VO_2max_ and resting metabolic rate.
		12			32.3 ± 7.1		SIT		All-out effort	3	20	120	
		6			37.5 ± 8.3		Control	No exercise					
Zhang et al. (2021)	11 females		21 ± 2	Obese	26.7 ± 3.4^a^		SIT		All-out effort	40	6	9	HIIT was effective for visceral fat loss, offering a feasible alternative to all-out SIT.
	12 females		20 ± 1		26.4 ± 4.1^a^		SIT		120% VO_2max_	--	60	90	
	12 females		20 ± 1		28.7 ± 2.3^a^		HIIT		90% VO_2max_	--	240	180	
	13 females		21 ± 2		28.9 ± 3.8^a^		Control	No exercise					

Minimal speed to elicit VO2max (vVO2max); Time to exhaustion at vVO_2_max (T_max_); Maximal Metabolic Steady State (MMSS); Maximal oxygen consumption (VO2max); Resting heart rate (RHR); Peak Oxygen Uptake (VO2peak); Peak power output (PPO); Increase ↑; Decrease ↓.

^a^ Indicates VO_2_peak.

Exercise modalities included both running and cycling protocols, with intensity levels ranging from 80% to 130% of VO_2_max or maximal sprint effort. SIT protocols generally involved short, “all-out” intervals of 6–40 repetitions lasting 6–60 s, whereas HIIT protocols included longer intervals of 3–16 repetitions lasting 60–240 s at submaximal intensities (typically ≥85% HRmax or VO_2_max). Recovery periods between intervals varied from 60 to 270 s, depending on the protocol.

Overall, SIT interventions were applied in 8 study arms, HIIT in 9 arms, and control groups in all studies. Across the included studies, intervention duration ranged from 4 to 12 weeks, with training frequency typically set at 2–4 sessions per week. Exercise intensity for HIIT protocols was generally between 80% and 100% of maximal heart rate or 85%–95% of VO_2_max, whereas SIT protocols consisted of repeated all-out sprints performed at ≥100% VO_2_max. The outcome assessments primarily focused on cardiorespiratory fitness (VO_2_max or VO_2_peak, expressed in mL·kg^−1^·min^−1^)) and body fat outcomes (reported mainly as body fat percentage). These standardized measures provided comparable endpoints across studies for meta-analytic evaluation of the training effects.

### 3.2 Comparative effects of SIT and HIIT on CRF

#### 3.2.1 The effects of SIT and HIIT on CRF compared to control

**FIGURE 2:**
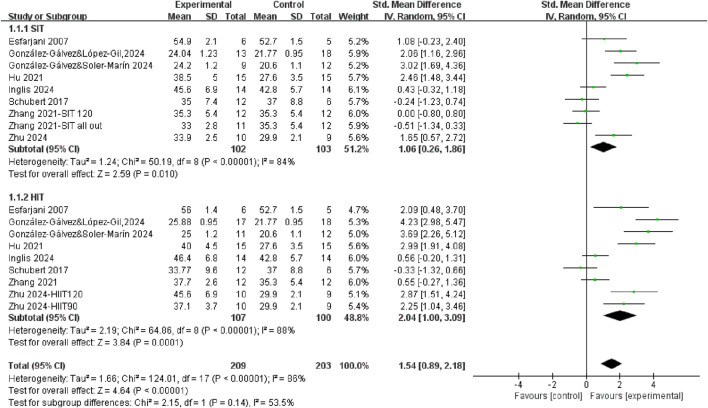
The effects of SIT and HIIT on CRF compared to control.

A total of 8 studies measured Vo2max or Vo2peak after SIT training or HIIT training compared with control group (Figure 2). Regardless of the training mode, there was significantly improved in CRF after training (SMD = 1.54, 95% CI = 0.89, 2.18, P < 0.00001), with a high level of heterogeneity (*I* ^2^ = 86%). Subgroup analysis revealed that both SIT and HIIT significantly improved CRF compared to control. Even though the individuals showed a moderate effect size after SIT training (SMD = 1.06, 95% CI = 0.26, 1.86, P = 0.01) while the HIIT group exhibited a large effect size (SMD = 2.04, 95% CI = 1.00, 3.09, P = 0.0001). However, the difference between SIT and HIIT subgroups was not statistically significant (p = 0.14), indicating that both training modalities were effective, with no clear superiority of one over the other. To further explore the comparative effectiveness between HIIT and SIT, we conducted a direct comparison in the following subgroup analysis.

#### 3.2.2 The effects of SIT and HIIT on CRF in different populations

**FIGURE 3:**
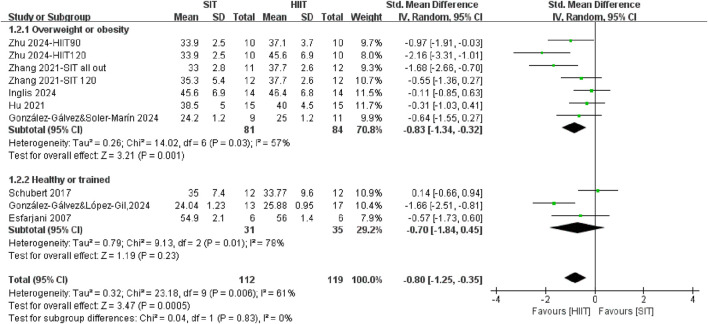
The effects of SIT and HIIT on CRF in different populations.

A subgroup meta-analysis was conducted to directly compare the effects of SIT training and HIIT training on CRF across different populations (Figure 3). In general, it demonstrated that HIIT was significantly more effective than SIT in improving CRF (SMD = −0.80, 95% CI = −1.25, −0.35, P = 0.0005), with moderate heterogeneity (*I* ^2^ = 61%). The analysis demonstrated a large effect size in CRF after HIIT training among individuals with overweight or obesity population (SMD = −0.97, 95% CI = −1.51, −0.43, P = 0.0004) compared to individuals with healthy or trained population, who showed no different in improving CRF after SIT training compared with HIIT training (SMD = −0.54, 95% CI = −1.36, 0.28, P = 0.20). However, the formal test for subgroup differences did not reach statistical significance (P = 0.39).

### 3.3 Comparative effects of SIT and HIIT on body fat mass

#### 3.3.1 The effects of SIT and HIIT on body fat mass compared to control

**FIGURE 4:**
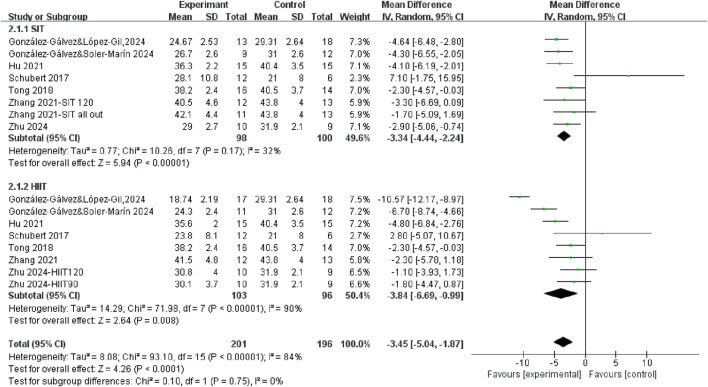
The effects of SIT and HIIT on body fat mass compared to control.

Seven studies measured fat mass differences after HIIT training and SIT training compared to control group (Figure 4). The results demonstrated that exercise interventions, regardless of training modality, led to a significant reduction in fat mass in the overall population (WMD = −3.45, 95% CI = −5.04, −1.87, P < 0.0001), with high heterogeneity (*I* ^2^ = 84%). Specifically, the fat mass percentage was significantly decreased after both SIT training (WMD = −3.34, 95% CI = −4.44, −2.24, P < 0.00001) and HIIT training (WMD = −3.84, 95% CI = −6.69, −0.99, P < 0.00001). However, the test for subgroup differences was not statistically significant (P = 0.75), suggesting that SIT and HIIT produced comparable effects in reducing fat mass.

#### 3.3.2 The effects of SIT and HIIT on body fat mass in different populations

**FIGURE 5:**
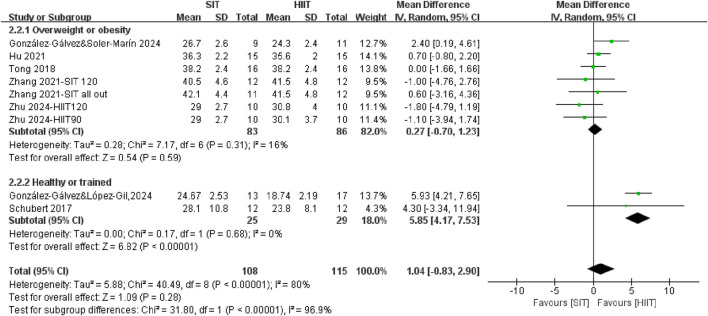
The effects of SIT and HIIT on body fat mass in different populations.

The meta-analysis revealed no statistically significant differences between SIT training and HIIT training interventions in terms of changes in body fat mass percentage (WMD = 1.04, 95% CI = −0.83, 2.90, P = 0.28) (Figure 5). However, stratification of the subgroups based on population characteristics yielded noteworthy findings. SIT appears to be more effective than HIIT in trained or healthy individuals for fat mass reduction (P < 0.00001). Although no significant difference was observed between HIIT and SIT interventions in individuals with overweight or obesity (WMD = 0.27, 95% CI = −0.70, 1.23, P = 0.59), SIT demonstrated greater efficacy than HIIT in reducing fat mass among trained or healthy individuals (WMD = 5.85, 95% CI = 4.17, 7.53, P < 0.00001). It suggested that the healthy level of population may modify the relative effectiveness of SIT training and HIIT training on fat mass.

## 4 Discussion

This meta-analysis demonstrated that both SIT and HIIT significantly improved CRF and reduced body fat mass when compared with control conditions. These findings are consistent with previous studies, which have highlighted the efficacy of both training modalities in enhancing cardiovascular health and improving body composition (Ko et al., 2025; Poon et al., 2024). Although CRF and body composition are not direct measures of health status, they remain important indicators of health-related physical fitness and exercise effectiveness. However, substantial heterogeneity was observed across the included studies. This variability may be attributed to differences in training frequency, intervention duration, exercise intensity, as well as participant characteristics such as sex, age, and baseline fitness level. This degree of heterogeneity suggests that the pooled estimates should be interpreted with caution, as differences in study design and participant characteristics may have influenced the magnitude of the observed effects.

The present study further explored these differences through subgroup analyses based on population health characteristics and directly compared the effectiveness of SIT and HIIT. The findings also revealed that individuals with different baseline fitness levels may respond differently to the two training modalities. However, these subgroup results should be interpreted with caution due to the limited number of studies and participants within each category. This not only reflects the distinct underlying physiological mechanisms of SIT and HIIT but also highlights the regulatory role of fitness level in determining training adaptations. Individuals with obesity may respond more favorably to the progressive and structured nature of HIIT, which typically involves prescribed intensity targets, controlled work-to-rest ratios, and gradual overload adjustments. In contrast, SIT generally consists of repeated all-out sprints of fixed duration without progressive modification (Buchheit and Laursen, 2013; MacInnis and Gibala, 2017). While healthy or trained individuals may derive greater benefit from SIT. These variations underscore the importance of considering both individual-level and protocol-level factors when interpreting training outcomes and tailoring exercise prescriptions. Nevertheless, these subgroup findings should be verified by future large, well-controlled studies.

### 4.1 Effects on CRF

The results of this meta-analysis indicated that both HIIT and SIT significantly improved CRF. Although the difference between the two modalities did not reach statistical significance, the observed trend suggested that HIIT might have offered greater overall benefits for improving CRF than SIT. Unlike previous meta-analyses that primarily focused on overall training effects, the present study provided novel insights by systematically comparing training responses across different population groups. However, further studies with larger sample sizes are required to confirm these observations. Although some recent studies compared HIIT and traditional aerobic training in terms of CRF improvements, they did not distinguish between participants with varying health statuses (Wewege et al., 2017). In contrast, our subgroup analyses clearly demonstrated that baseline health level significantly influenced the differential effects of HIIT and SIT. Specifically, the superiority of HIIT became more apparent among individuals with overweight or obesity, whereas no significant difference was observed between HIIT and SIT in healthy or trained populations. These findings indicated that population fitness status might have moderated training responsiveness and underscored the importance of tailoring exercise interventions based on baseline fitness levels.

The differential responses to interval training may have stemmed from variations in interval duration, intensity, and metabolic pathways. Compared to SIT, which relied more on anaerobic metabolism and neuromuscular activation through very short “all-out” efforts (Atakan et al., 2022), HIIT typically involved prolonged intervals near VO_2_max, activating aerobic metabolic pathways that enhanced mitochondrial biogenesis, capillary density, and cardiac output, thereby eliciting comprehensive cardiorespiratory adaptations (MacInnis and Gibala, 2017). However, similar physiological adaptations, including increased mitochondrial protein expression and capillary growth, have also been observed following SIT interventions (Cocks et al., 2016; Mitchell et al., 2019). Moreover, a recent study supported that HIIT performed above 85% HRmax was more effective at eliciting significant increases in CRF (Sloth et al., 2013). The differential effects of HIIT and SIT may also relate to differences in glycolytic contribution, recovery capacity, and muscle fiber recruitment (MacInnis and Gibala, 2017). SIT imposes greater anaerobic and neuromuscular demands, whereas HIIT elicits stronger oxidative and mitochondrial adaptations, which may favor individuals with lower baseline fitness or obesity (Bishop et al., 2019). These mechanisms are consistent with recent umbrella and meta-analytic evidence (Poon et al., 2024; Liu et al., 2025).

These physiological characteristics may have explained why HIIT was found to be more effective than SIT in improving CRF among individuals with overweight or obesity in the present meta-analysis. Individuals with obesity, due to their lower baseline VO_2_max and greater capacity for physiological adaptation, may have been more responsive to the structured, high-intensity, and moderate-duration nature of HIIT (Wen et al., 2019). Although SIT was capable of eliciting strong metabolic responses, it may have posed challenges in terms of tolerability and safety for individuals with compromised cardiorespiratory function or lower exercise adherence, potentially limiting its long-term effectiveness (Atakan et al., 2021).

In contrast, there was similar CRF improvement after HIIT or SIT between healthy or trained individuals. In these populations, the high-intensity, time-efficient nature of SIT may still provide sufficient cardiovascular stimulation to induce adaptations, thereby achieving the same improvement as HIIT (Gillen and Gibala, 2014). Therefore, the observed equivalence in CRF improvements between HIIT and SIT in healthy individuals may have reflected a “ceiling effect,” in which the adaptive potential was limited by already well-developed cardiorespiratory function (Bouchard et al., 2012). Overall, SIT remains a time-efficient alternative and may be preferable for individuals with limited exercise time in health or trained. These findings underscore the importance of tailoring interval training modalities to individuals’ baseline fitness levels and physiological profiles.

### 4.2 Effects on body composition

This meta-analysis revealed that both SIT and HIIT interventions significantly reduced body fat mass compared to control conditions, with no statistically significant difference between the two modalities in the overall analysis. These findings indicated that both training approaches were effective in improving body composition (Wewege et al., 2017; Keating et al., 2017). A recent large-scale analysis involving 36 randomized controlled trials also confirmed that both HIIT and SIT reduced body fat percentage and mass in overweight individual (Liu et al., 2025). However, our subgroup analyses by population health status yielded important difference. Specifically, SIT appeared to be more effective than HIIT in reducing fat mass among healthy or trained individuals, while no significant difference was observed in individuals with overweight or obesity. This suggests that the effectiveness of training modalities is not uniform across populations, and baseline health status may act as a moderator of intervention outcomes.

The superior performance of SIT in healthy or trained populations may reflect their higher tolerance for supramaximal efforts and greater metabolic flexibility. SIT induces potent anaerobic and catecholaminergic responses, which in these individuals may enhance fat oxidation and hormonal signaling, including growth hormone and norepinephrine release, both of which are linked to lipolysis (Bouchard et al., 2012). Moreover, the time efficiency of SIT, which required as little as half the total duration of HIIT, had been validated in multiple studies (Tong et al., 2018; Gillen et al., 2016), reinforcing its practicality for time-constrained individuals. Nonetheless, these advantages may not generalize to individuals with overweight or obesity, who may struggle to sustain the maximal efforts required for effective SIT. Reduced tolerance, impaired recovery capacity, and cardiometabolic limitations may diminish training intensity and compromise the expected fat-loss outcomes. While both training types yielded similar effects in this group, HIIT’s structured nature may offer a more feasible and sustainable pathway to fat reduction. Prior studies have emphasized HIIT’s acceptability and safety in clinical populations (Keating et al., 2017; Khodadadi et al., 2023), aligning with our findings.

While the current evidence base supports the overall effectiveness of interval training, methodological variability across studies must be acknowledged. Differences in training duration, exercise modality (cycling vs. running), supervision, and dietary control may confound the comparability of outcomes. For example, many studies included in previous reviews did not distinguish between HIIT and SIT with sufficient clarity (Wewege et al., 2017), limiting the strength of direct comparisons. Considerable heterogeneity (*I* ^2^ > 80%) was observed in some analyses. Although additional subgroup analyses were considered, they were not feasible due to the limited number of studies, inconsistent reporting, and overlapping interventions. Nevertheless, effect directions were consistent across studies, suggesting that heterogeneity mainly reflected differences in participant age and baseline fitness rather than inconsistencies in outcomes. In addition, sensitivity analysis showed that the pooled results remained stable after sequential exclusion of individual studies.

Furthermore, the observed subgroup effects, while statistically robust, may reflect selection biases or variations in baseline body composition rather than training modality alone. In overall, our findings suggest that both SIT and HIIT are effective strategies for reducing body fat mass, but their relative efficacy may depend on the health and fitness profiles of participants. SIT may be better suited for healthy individuals seeking efficient fat loss, while HIIT may offer a more accessible and tolerable approach for overweight or clinical populations. These results highlight the importance of individualized program design and call for future studies to standardize intervention protocols and explore underlying physiological mechanisms across diverse populations.

## 5 Conclusion

This meta-analysis demonstrated that both HIIT and SIT effectively improved cardiorespiratory fitness and reduced body fat mass. HIIT appeared more effective for improving CRF in individuals with overweight or obesity, while SIT showed greater fat-reduction benefits in healthy or trained populations. These findings highlight the importance of tailoring interval training strategies based on baseline fitness status. Given its time efficiency, SIT may be particularly suitable for trained individuals, whereas HIIT may offer a more accessible option for lower-fit populations. Future research should focus on protocol standardization and long-term outcomes across different groups.

## Data Availability

The original contributions presented in the study are included in the article/Supplementary Material, further inquiries can be directed to the corresponding authors.

## References

[B1] Amaro-Gahete F. J. Sanchez-Delgado G. Ara I. J R. R. (2019). Cardiorespiratory fitness may influence metabolic inflexibility during exercise in Obese persons. J. Clin. Endocrinol. Metab. 104, 5780–5790. 10.1210/jc.2019-01225 31322652

[B2] Atakan M. M. Li Y. Koşar Ş N. Turnagöl H. H. Yan X. (2021). Evidence-based effects of high-intensity interval training on exercise capacity and health: a review with historical perspective. Int. J. Environ. Res. Public Health 18, 7201. 10.3390/ijerph18137201 34281138 PMC8294064

[B3] Atakan M. M. Guzel Y. Shrestha N. Kosar S. N. Grgic J. Astorino T. A. (2022). Effects of high-intensity interval training (HIIT) and sprint interval training (SIT) on fat oxidation during exercise: a systematic review and meta-analysis. Br. J. Sports Med. 56, 988–996. 10.1136/bjsports-2021-105181 35859145

[B4] Bishop D. J. Botella J. Genders A. J. Lee M. J. Saner N. J. Kuang J. (2019). High-intensity exercise and mitochondrial biogenesis: current controversies and future research directions. Physiol. (Bethesda) 34, 56–70. 10.1152/physiol.00038.2018 30540234

[B5] Bouchard C. Blair S. N. Church T. S. Earnest C. P. Hagberg J. M. Häkkinen K. (2012). Adverse metabolic response to regular exercise: is it a rare or common occurrence? PLoS One 7, e37887. 10.1371/journal.pone.0037887 22666405 PMC3364277

[B6] Boullosa D. Dragutinovic B. Feuerbacher J. F. Benítez-Flores S. Coyle E. F. Schumann M. (2022). Effects of short sprint interval training on aerobic and anaerobic indices: a systematic review and meta-analysis. Scand. J. Med. Sci. Sports 32, 810–820. 10.1111/sms.14133 35090181

[B7] Buchheit M. Laursen P. B. (2013). High-intensity interval training, solutions to the programming puzzle: part I: cardiopulmonary emphasis. Sports Med. 43, 313–338. 10.1007/s40279-013-0029-x 23539308

[B8] Bull F. C. Al-Ansari S. S. Biddle S. Borodulin K. Buman M. P. Cardon G. (2020). World Health Organization 2020 guidelines on physical activity and sedentary behaviour. Br. J. Sports Med. 54, 1451–1462. 10.1136/bjsports-2020-102955 33239350 PMC7719906

[B9] Cocks M. Shaw C. S. Shepherd S. O. Fisher J. P. Ranasinghe A. Barker T. A. (2016). Sprint interval and moderate-intensity continuous training have equal benefits on aerobic capacity, insulin sensitivity, muscle capillarisation and endothelial eNOS/NAD(P)Hoxidase protein ratio in obese men. J. Physiol. 594, 2307–2321. 10.1113/jphysiol.2014.285254 25645978 PMC4933110

[B10] Cooke M. B. Deasy W. Ritenis E. J. Wilson R. A. Stathis C. G. (2022). Effects of intermittent energy restriction alone and in combination with sprint interval training on body composition and cardiometabolic biomarkers in individuals with overweight and obesity. Int. J. Environ. Res. Public Health 19, 7969. 10.3390/ijerph19137969 35805627 PMC9265557

[B11] Esfarjani F. Laursen P. B. (2007). Manipulating high-intensity interval training: effects on VO2max, the lactate threshold and 3000 m running performance in moderately trained males. J. Sci. Med. Sport 10, 27–35. 10.1016/j.jsams.2006.05.014 16876479

[B12] Garthwaite T. Sjöros T. Laine S. Koivumäki M. Vähä-Ypyä H. Verho T. (2024). Sedentary time associates detrimentally and physical activity beneficially with metabolic flexibility in adults with metabolic syndrome. Am. J. Physiol. Endocrinol. Metab. 326, E503–e514. 10.1152/ajpendo.00338.2023 38416072 PMC11194051

[B13] Gillen J. B. Gibala M. J. (2014). Is high-intensity interval training a time-efficient exercise strategy to improve health and fitness? Appl. Physiol. Nutr. Metab. 39, 409–412. 10.1139/apnm-2013-0187 24552392

[B14] Gillen J. B. Martin B. J. MacInnis M. J. Skelly L. E. Tarnopolsky M. A. Gibala M. J. (2016). Twelve weeks of sprint interval training improves indices of cardiometabolic health similar to traditional endurance training despite a five-fold lower exercise volume and time commitment. PLoS One 11, e0154075. 10.1371/journal.pone.0154075 27115137 PMC4846072

[B15] González-Gálvez N. López-Gil J. F. Espeso-Garcia A. Abenza-Cano L. Mateo-Orcajada A. Vaquero-Cristóbal R. (2024a). Effectiveness of high intensity and sprint interval training on metabolic biomarkers, body composition, and physical fitness in adolescents: randomized controlled trial. Front. Public Health 12, 1425191. 10.3389/fpubh.2024.1425191 39157534 PMC11328537

[B16] González-Gálvez N. Soler-Marín A. Abelleira-Lamela T. Abenza-Cano L. Mateo-Orcajada A. Vaquero-Cristóbal R. (2024b). Eight weeks of high-intensity interval vs. sprint interval training effects on overweight and obese adolescents carried out during the cool-down period of physical education classes: randomized controlled trial. Front. Public Health 12, 1394328. 10.3389/fpubh.2024.1394328 38746000 PMC11092892

[B17] Hebisz P. Hebisz R. Murawska-Ciałowicz E. Zatoń M. (2019). Changes in exercise capacity and serum BDNF following long-term sprint interval training in well-trained cyclists. Appl. Physiol. Nutr. Metab. 44, 499–506. 10.1139/apnm-2018-0427 30286300

[B18] Hov H. Wang E. Lim Y. R. Trane G. Hemmingsen M. Hoff J. (2023). Aerobic high-intensity intervals are superior to improve V̇O(2max) compared with sprint intervals in well-trained men. Scand. J. Med. Sci. Sports 33, 146–159. 10.1111/sms.14251 36314990 PMC10099854

[B19] HsuK. J. ChienK. Y. TsaiS. C. TsaiY. S. LiaoY. H. ChenJ. J. (2021). Effects of exercise alone or in combination with high-protein diet on muscle function, aerobic capacity, and physical function in middle-aged Obese adults: a randomized controlled trial. J. Nutr. Health Aging 25, 727–734. 10.1007/s12603-021-1599-1 34179925

[B20] Hu M. Kong Z. Sun S. Zou L. Shi Q. Chow B. C. (2021). Interval training causes the same exercise enjoyment as moderate-intensity training to improve cardiorespiratory fitness and body composition in young Chinese women with elevated BMI. J. Sports Sci. 39, 1677–1686. 10.1080/02640414.2021.1892946 33634738

[B21] Inglis E. C. Iannetta D. Rasica L. Mackie M. Z. Keir D. A. Macinnis M. J. (2024). Heavy-Severe-and extreme-but not moderate-intensity exercise increase V̇o 2max and thresholds after 6 wk of training. Med. Sci. Sports Exerc 56, 1307–1316. 10.1249/MSS.0000000000003406 38376995

[B22] Keating S. E. Johnson N. A. Mielke G. I. Coombes J. S. (2017). A systematic review and meta-analysis of interval training *versus* moderate-intensity continuous training on body adiposity. Obes. Rev. 18, 943–964. 10.1111/obr.12536 28513103

[B23] Khodadadi F. Bagheri R. Negaresh R. Moradi S. Nordvall M. Camera D. M. (2023). The effect of high-intensity interval training type on body fat percentage, fat and fat-free mass: a systematic review and meta-analysis of randomized clinical trials. J. Clin. Med. 12, 2291. 10.3390/jcm12062291 36983289 PMC10054577

[B24] Ko J. M. So W. Y. Park S. E. (2025). Narrative review of high-intensity interval training: positive impacts on cardiovascular health and disease prevention. J. Cardiovasc Dev. Dis. 12, 158. 10.3390/jcdd12040158 40278218 PMC12027975

[B25] Kodama S. Saito K. Tanaka S. Maki M. Yachi Y. Asumi M. (2009). Cardiorespiratory fitness as a quantitative predictor of all-cause mortality and cardiovascular events in healthy men and women: a meta-analysis. JAMA 301, 2024–2035. 10.1001/jama.2009.681 19454641

[B26] Liu H. Li Q. Yang W. Poon E. T. Liu H. Bao D. (2025). Effects of HIIT and sprint interval training on adiposity in overweight adults: a meta-analysis. Int. J. Sports Med. 10.1055/a-2559-8063 40097160

[B27] MacInnis M. J. Gibala M. J. (2017). Physiological adaptations to interval training and the role of exercise intensity. J. Physiol. 595, 2915–2930. 10.1113/JP273196 27748956 PMC5407969

[B28] Mitchell E. A. Martin N. R. W. Turner M. C. Taylor C. W. Ferguson R. A. (2019). The combined effect of sprint interval training and postexercise blood flow restriction on critical power, capillary growth, and mitochondrial proteins in trained cyclists. J. Appl. Physiol. 126, 51–59. 10.1152/japplphysiol.01082.2017 30335575

[B29] Murray C. J. L. Aravkin A. Y. Zheng P. Abbafati C. Abbas K. M. Abbasi-Kangevari M. (2020). Global burden of 87 risk factors in 204 countries and territories, 1990–2019: a systematic analysis for the global burden of disease study 2019. Lancet 396, 1223–1249. 10.1016/S0140-6736(20)30752-2 33069327 PMC7566194

[B30] Oliveira J. Gentil P. Naves J. P. Souza Filho L. F. Silva L. Zamunér A. R. (2022). Effects of high intensity interval training *versus* sprint interval training on cardiac autonomic modulation in healthy women. Int. J. Environ. Res. Public Health 19, 12863. 10.3390/ijerph191912863 36232163 PMC9566246

[B31] Paquette M. Bieuzen F. Billaut F. (2021). The effect of HIIT vs. SIT on muscle oxygenation in trained sprint kayakers. Eur. J. Appl. Physiol. 121, 2743–2759. 10.1007/s00421-021-04743-z 34145486

[B32] Pinho C. D. F. Bagatini-Ph D. N. Lisboa S. D. C. Mello J. B. Cunha G. D. S. (2024). Effects of different supervised and structured physical exercise on the physical fitness trainability of children and adolescents: a meta-analysis and meta-regression: physical fitness trainability in children and adolescents' health. BMC Pediatr. 24, 798. 10.1186/s12887-024-04929-2 39639233 PMC11619429

[B33] Poon E.T.-C. Li H.-Y. Little J. P. Wong S.H.-S. Ho R.S.-T. (2024). Efficacy of interval training in improving body composition and adiposity in apparently healthy adults: an umbrella review with meta-analysis. Sports Med. 54, 2817–2840. 10.1007/s40279-024-02070-9 39003682 PMC11560999

[B34] Salus M. Tillmann V. Remmel L. Unt E. Mäestu E. Parm Ü. (2022). Effect of supervised sprint interval training on cardiorespiratory fitness and body composition in adolescent boys with obesity. J. Sports Sci. 40, 2010–2017. 10.1080/02640414.2022.2125199 36126151

[B35] Schubert M. M. Clarke H. E. Seay R. F. Spain K. K. (2017). Impact of 4 weeks of interval training on resting metabolic rate, fitness, and health-related outcomes. Appl. Physiol. Nutr. Metab. 42, 1073–1081. 10.1139/apnm-2017-0268 28633001

[B36] Sloth M. Sloth D. Overgaard K. Dalgas U. (2013). Effects of sprint interval training on VO2max and aerobic exercise performance: a systematic review and meta-analysis. Scand. J. Med. Sci. Sports 23, e341–e352. 10.1111/sms.12092 23889316

[B37] Sun S. Zhang H. Kong Z. Shi Q. Tong T. K. Nie J. (2019). Twelve weeks of low volume sprint interval training improves cardio-metabolic health outcomes in overweight females. J. Sports Sci. 37, 1257–1264. 10.1080/02640414.2018.1554615 30563431

[B38] Tong T. K. Zhang H. Shi H. Liu Y. Ai J. Nie J. (2018). Comparing time efficiency of sprint vs. high-intensity interval training in reducing abdominal visceral fat in Obese young women: a randomized, controlled trial. Front. Physiol. 9, 1048. 10.3389/fphys.2018.01048 30123136 PMC6085472

[B39] Wen D. Utesch T. Wu J. Robertson S. Liu J. Hu G. (2019). Effects of different protocols of high intensity interval training for VO(2)max improvements in adults: a meta-analysis of randomised controlled trials. J. Sci. Med. Sport 22, 941–947. 10.1016/j.jsams.2019.01.013 30733142

[B40] Wewege M. van den Berg R. Ward R. E. Keech A. (2017). The effects of high-intensity interval training vs. moderate-intensity continuous training on body composition in overweight and obese adults: a systematic review and meta-analysis. Obes. Rev. 18, 635–646. 10.1111/obr.12532 28401638

[B41] Zhang H. Tong T. K. Kong Z. Shi Q. Liu Y. Nie J. (2021). Exercise training-induced visceral fat loss in obese women: the role of training intensity and modality. Scand. J. Med. Sci. Sports 31, 30–43. 10.1111/sms.13803 32789898

[B42] Zhu X. Jiao J. Liu Y. Li H. Zhang H. (2024). The release of lipolytic hormones during various high-intensity interval and moderate-intensity continuous training regimens and their effects on fat loss. J. Sports Sci. Med. 23, 559–570. 10.52082/jssm.2024.559 39228779 PMC11366854

