# Supplementary material for: Comparative effects of high-intensity and sprint interval training on cardiorespiratory fitness and body composition: a systematic review with meta-analysis
Source: Front Physiol. 2025 Nov 11;16:1668326. doi: 10.3389/fphys.2025.1668326 (PMC12643836; doi:10.3389/fphys.2025.1668326)
Supplement: Supplementary file 1 [file Supplementaryfile1.docx]

Supplementary Material


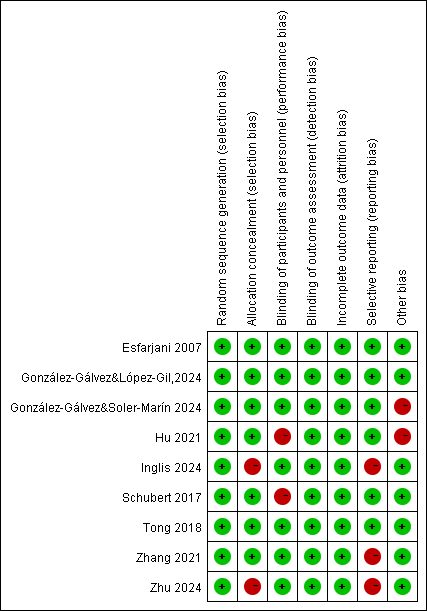


**Supplementary Figure S1.** Traffic-light plot illustrating domain-level risk-of-bias assessment for each included randomized trial.


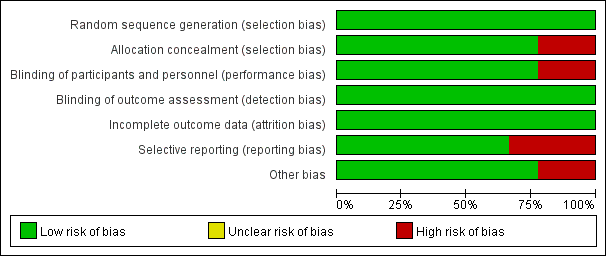


**Supplementary Figure S1.** Summary graph showing the proportion of studies rated low, some concerns, or high risk of bias across each risk-of-bias assessment domain.
