# Supplementary material for: Comparative effects of high-intensity and sprint interval training on cardiorespiratory fitness and body composition: a systematic review with meta-analysis
Source: Front Physiol. 2025 Nov 11;16:1668326. doi: 10.3389/fphys.2025.1668326 (PMC12643836; doi:10.3389/fphys.2025.1668326)
Supplement: Supplementary file 2 [file Table1.docx]

| **Database** | **Search string** | **Notes / Filters** |
| --- | --- | --- |
| **PubMed** | (((“high-intensity interval training”[Title/Abstract] OR HIIT[Title/Abstract]) OR (“sprint interval training”[Title/Abstract] OR SIT[Title/Abstract])) AND (“VO2max”[Title/Abstract] OR “VO₂max”[Title/Abstract] OR “cardiorespiratory fitness”[Title/Abstract] OR “aerobic capacity”[Title/Abstract]) AND (“body composition”[Title/Abstract] OR “body fat”[Title/Abstract] OR “fat mass”[Title/Abstract])) AND (randomized controlled trial[Publication Type] OR RCT[Title/Abstract]) | No language or date limits applied; filters: Humans |
| **Embase** | (‘high intensity interval training’:ab,ti OR HIIT:ab,ti) OR (‘sprint interval training’:ab,ti OR SIT:ab,ti) AND (‘VO2max’:ab,ti OR ‘cardiorespiratory fitness’:ab,ti OR ‘aerobic capacity’:ab,ti) AND (‘body composition’:ab,ti OR ‘fat mass’:ab,ti OR ‘body fat percentage’:ab,ti) AND (‘randomized controlled trial’/exp OR RCT:ab,ti) | No language or date limits applied |
| **Web of Science (Core Collection)** | TS= (“high-intensity interval training” OR HIIT OR “sprint interval training” OR SIT) AND TS= (“VO2max” OR “cardiorespiratory fitness” OR “aerobic capacity”) AND TS= (“body composition” OR “body fat” OR “fat mass”) AND TS= (randomized OR controlled OR trial) | Timespan: All years; Language: All; Document type: Article |
| **Cochrane Library** | (“high-intensity interval training” OR HIIT OR “sprint interval training” OR SIT) in Title, Abstract, Keyword AND (“VO2max” OR “cardiorespiratory fitness” OR “aerobic capacity”) in Title, Abstract, Keyword AND (“body composition” OR “body fat” OR “fat mass”) in Title, Abstract, Keyword | Database: CENTRAL; No date restriction |
| **SPORTDiscus** | (DE “high-intensity interval training” OR TI HIIT OR AB “high-intensity interval training” OR “sprint interval training” OR SIT) AND (DE “cardiorespiratory fitness” OR TI “VO2max” OR AB “aerobic capacity”) AND (DE “body composition” OR “fat mass” OR “body fat”) | No date or language restrictions; Database filter: Peer-reviewed only |

**Supplementary Table S1.** Complete search strategy for all databases.
