# Supplementary material for: Comparative effects of high-intensity and sprint interval training on cardiorespiratory fitness and body composition: a systematic review with meta-analysis
Source: Front Physiol. 2025 Nov 11;16:1668326. doi: 10.3389/fphys.2025.1668326 (PMC12643836; doi:10.3389/fphys.2025.1668326)
Supplement: Supplementary file 3 [file Supplementaryfile2.docx]

**Funnel plot**


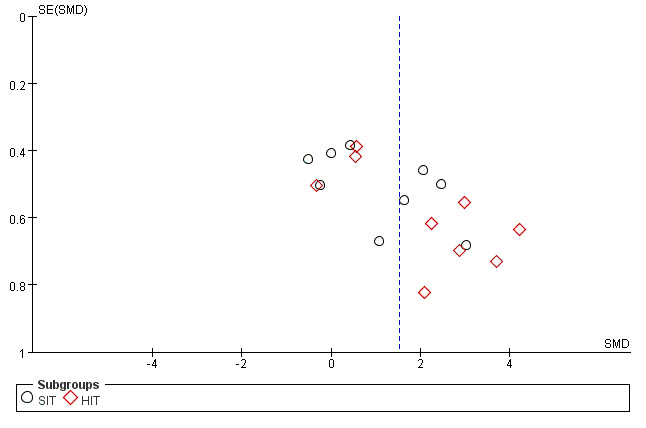
 **Supplementary Figure S3.** Funnel plot assessing potential publication bias for CRF.

Funnel plot showing the distribution of studies according to standardized mean difference (SMD) and standard error (SE).

Circles represent SIT interventions and diamonds represent HIIT interventions.

The plot appears symmetrical around the pooled effect estimate, suggesting no substantial publication bias for CRF outcomes.


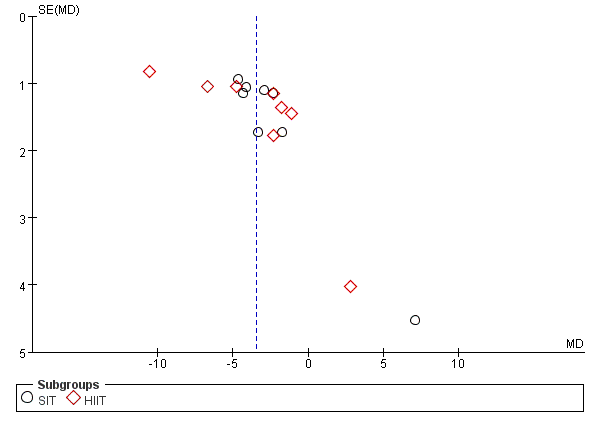


**Supplementary Figure S4.** Funnel plot assessing potential publication bias for body fat mass.

Funnel plot showing the distribution of studies according to mean difference (MD) and standard error (SE).

Circles represent SIT interventions and diamonds represent HIIT interventions.

The plot shows an approximately symmetrical distribution of studies, indicating minimal publication bias across trials for body fat outcomes.
